# Alzheimer's Disease Related Markers, Cellular Toxicity and Behavioral Deficits Induced Six Weeks after Oligomeric Amyloid-β Peptide Injection in Rats

**DOI:** 10.1371/journal.pone.0053117

**Published:** 2013-01-02

**Authors:** Charleine Zussy, Anthony Brureau, Emeline Keller, Stéphane Marchal, Claire Blayo, Brice Delair, Guy Ixart, Tangui Maurice, Laurent Givalois

**Affiliations:** 1 Molecular Mechanisms in Neurodegenerative Dementia Laboratory, Inserm U710, Montpellier, France; 2 University of Montpellier 2, Montpellier, France; 3 EPHE, Paris, France; Nathan Kline Institute and New York University School of Medicine, United States of America

## Abstract

Alzheimer’s disease (AD) is a neurodegenerative pathology associated with aging characterized by the presence of senile plaques and neurofibrillary tangles that finally result in synaptic and neuronal loss. The major component of senile plaques is an amyloid-β protein (Aβ). Recently, we characterized the effects of a single intracerebroventricular (icv) injection of Aβ fragment (25–35) oligomers (oAβ_25–35_) for up to 3 weeks in rats and established a clear parallel with numerous relevant signs of AD. To clarify the long-term effects of oAβ_25–35_ and its potential role in the pathogenesis of AD, we determined its physiological, behavioral, biochemical and morphological impacts 6 weeks after injection in rats. oAβ_25–35_ was still present in the brain after 6 weeks. oAβ_25–35_ injection did not affect general activity and temperature rhythms after 6 weeks, but decreased body weight, induced short- and long-term memory impairments, increased corticosterone plasma levels, brain oxidative (lipid peroxidation), mitochondrial (caspase-9 levels) and reticulum stress (caspase-12 levels), astroglial and microglial activation. It provoked cholinergic neuron loss and decreased brain-derived neurotrophic factor levels. It induced cell loss in the hippocampic CA subdivisions and decreased hippocampic neurogenesis. Moreover, oAβ_25–35_ injection resulted in increased APP expression, Aβ_1–42_ generation, and increased Tau phosphorylation. In conclusion, this *in vivo* study evidenced that the soluble oligomeric forms of short fragments of Aβ, endogenously identified in AD patient brains, not only provoked long-lasting pathological alterations comparable to the human disease, but may also directly contribute to the progressive increase in amyloid load and Tau pathology, involved in the AD physiopathology.

## Introduction

Alzheimer’s disease (AD) is the most common cause of dementia in the elderly and is characterized by a progressive impairment in cognitive functions, resulting from synapse and nerve cell destruction in the brain. AD symptoms include memory loss, alteration of the individual’s personality and failure to communicate or perform routine tasks. The histopathological hallmarks of AD include the presence of extracellular senile plaques, intracellular neurofibrillary tangles (NFT), reduction and dysfunction of synapses, neuronal death and reduction in overall brain volume. Senile plaques are composed of insoluble extracellular aggregates consisting mainly of amyloid-β (Aβ) peptides, which are generated by enzymatic cleavages of the amyloid precursor protein (APP), while NFT are the result of hyper- and abnormal phosphorylation of the microtubule-stabilizing protein Tau [1], [2].

There is no doubt that progressive Aβ accumulation contributes to AD. A correlation between the total amount of Aβ in human brain and cognitive decline indicates that the amount of Aβ, but not necessarily plaque formation, is important for AD progression [3], [4]. Transgenic APP mice demonstrate cognitive decline before plaque formation [5], and soluble oligomers can inhibit cognitive function [6] and long-term potentiation [7], [8]. In fact, it is possible that extracellular amyloid deposits are only one aspect of the larger pathological cascade and an indirect consequence of possible protective responses intended to sequester toxic soluble Aβ oligomers [4]. The degree of dementia in AD correlates better with Aβ assayed biochemically, than with the histologically determined number of plaque. The concentration of soluble Aβ species, which cannot be detected through an immunohistochemical analysis, appears to be more closely correlated with cognitive deficits [3], [4]. In fact, Aβ deposits may not even carry the most aggressive toxicity, but instead represent a reserve of toxicity from where toxic oligomeric fragments could be released [9]–[11].

The soluble Aβ oligomers observed in AD patients contain Aβ in its most predominant sequences: Aβ_1–40_ or Aβ_1–42_ [3], [11]. Nevertheless, they also contain peptides with shorter sequences such as N-truncated amyloid-β oligomers [12], [13]: Aβ_25–35_ or Aβ_25–35/40_ [14]–[16]. Aβ_25–35_ (GSNKGAIIGLM) can be produced in AD patients by enzymatic cleavage of Aβ_1–40_ [14], [15]. This Aβ peptide includes extracellular and transmembrane residues that have been reported to represent a biologically active region of Aβ [17]–[19] and to contain the highly hydrophobic region forming stable aggregations [18]. Interest in this undecapeptide, which itself shows a β-sheet structure [18], [20], has grown over the last decade, mainly because it induces neurite atrophy, neuronal cell death, synaptic loss, as well as synaptic plasticity and memory deficits in a similar way to Aβ_1–40_ and Aβ_1–42_ [21], but with better solubility and efficiency [22], [23].

Most studies in rodents have examined the effects of Aβ_25–35_ 1, 2 and 3 weeks after its icv injection [20], [24]–[35]. Only one study continued the investigation up to 6 months after icv injection of Aβ_25–35_ and found long-lasting memory deficits [34].

Therefore, to clarify the long-term effects of a single icv injection of Aβ_25–35_ oligomers (oAβ_25–35_) and to evaluate a potential impact of such short Aβ fragment in the progression of AD, we conducted a study to determine its behavioral, physiological, biochemical and morphological impacts in healthy adult male rats, 6 weeks after a single icv injection, including changes in APP processing, Aβ_1–42_ generation and Tau hyperphosphorylation.

## Methods

### Animals

Adult male Sprague-Dawley rats (Depré, France) weighing 280–300 g at the beginning of the experiments were housed for 1 week before experiments under standard laboratory conditions (12 h/12 h light/dark cycle with lights on at 7:00 AM; 21±1°C, food and water *ad libitum*). The animals were treated in accordance with the European Community Council Directive (EEC/86/609). The Animal Welfare Committee at the University of Montpellier 2 approved all protocols and all efforts were made to minimize the number of animals used and potential pain and distress. All surgery was performed under Ketamine/Xylazine mixture, and all efforts were made to minimize suffering. All experiments were performed in conscious rats between 9:00 AM and 2:00 PM, i.e. during the diurnal trough of the circadian rhythm.

### Amyloid-β Peptide

Aβ_25–35_ and scrambled Aβ_25–35_ peptide (NeoMPS, France) were dissolved in sterile bidistilled water at a concentration of 1 µg/µl (soluble form) and stored at −20°C. Aβ_25–35_ and scrambled peptides were aggregated by *in vitro* incubation at 37°C for 4 days [26], to obtain a solution of Aβ_25–35_ oligomers (oAβ_25–35_).

**Figure 1:**
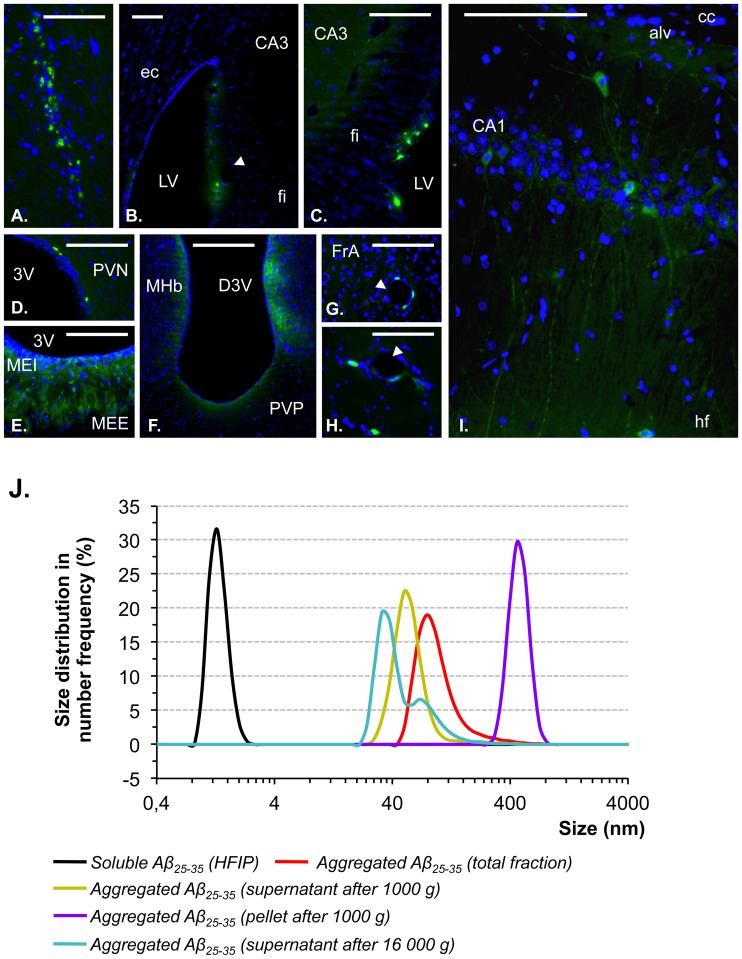
Brain localization of Aβ_25–35_ and particle characterization of Aβ_25–35_ solutions A–I. Localization within brain structures of oAβ_25–35_-HLF, determined 6 weeks after its icv injection (10 µg/rat). oAβ_25–35_-HLF was visualized in green, while the nucleus was counterstained with DAPI (blue labeling). Abbreviations: 3V: third ventricle; alv: alveus of the hippocampus; CA1: field CA1 of hippocampus; CA3: field CA3 of the hippocampus; cc: corpus callosum; D3V: dorsal third ventricle; ec: external capsule; fi: fimbria of the hippocampus; FrA: frontal association cortex; hf: hippocampal fissure; LV: lateral ventricle; MEE: median eminence, external part; MEI: median eminence, internal part; MHb: medial habenular nucleus; PVN: paraventricular hypothalamic nucleus; PVP: paraventricular thalamic nucleus, posterior part. Arrowhead: blood vessel. Scale bar  = 100 µm. **J.** Particle size distribution of the different fractions of Aβ_25–35_ solution (1 µg/µl) was determined by PCS at 25°C. Samples were prepared as described in the materials and methods section. Black curve: Aβ_25–35_ peptide dissolved in hexafluoroisopropanol (HFIP); Red curve: solution of aggregated Aβ_25–35_ peptide; Green curve: supernatant of aggregated Aβ_25–35_ peptide centrifuged at 1 000 g; Purple curve: re-suspended pellet of aggregated Aβ_25–35_ peptide obtained after centrifugation at 1 000 g; Blue curve: supernatant of aggregated Aβ_25–35_ peptide centrifuged at 16 000 g. Data were analyzed using a Zetasizer software 6.01 and expressed as size frequency distribution (%) in function of particles size (nm).

To evaluate the size of particles induced after the 4 days incubation at 37°C, different fractions (detailed in figure 1) were evaluated by photon correlation spectroscopy (PCS) using a Nanosizer (Zetasizer Nano Series ZS, Malvern Instruments, UK) with a laser light wavelength at 632.8 nm and a scattering angle of 173 degrees. The particle size (nm) was measured at 25°C using disposable microcuvettes (Malvern Instruments). The correlation times were defined on 10 s per run and a total of 13 runs was made per measurement. Before measurements, each sample was diluted if necessary in water to avoid multiple diffusion phenomena during PCS measurements. Results were analyzed using a Zetasizer software 6.01, experimental data were assessed by NNLS algorithm. For this calculation, the dispersant viscosity was taken as 0.89 mPa at 25°C and the refractive index as 1.33. Size distributions were expressed as size frequency distribution (%) in function of particles size (nm). As control, monomeric form of Aβ_25–35_ peptide was obtained by dilution in hexafluoroisopropanol (HFIP, Sigma-Aldrich, France).

### Experimental Procedures

Animals were divided into three groups. One group was left undisturbed (control rats), a second group received an icv injection of incubated scrambled peptide (10 µg/rat) and a third group received an icv injection of oAβ_25–35_ peptide (10 µg/rat) [25]. For icv injection through a Hamilton syringe (VWR, France), the animals were anesthetized with an intramuscular injection of 0.2 ml of a mixture of Ketamine hydrochloride (80 mg/kg b.w.) and Xylazine (10 mg/kg b.w.). They were then stereotaxically injected directly into the lateral ventricles at coordinates (AP: −1 mm, L: ±1.5 mm, and DV: −3.5 mm) according to Paxinos and Watson [35].

### Locomotor Activity and Body Temperature Variations

The day of the icv injection and during the same anesthetic session, a single telemetric transmitter (PhysioTel, TA 10TA-F40; DSI, USA) was implanted intra-peritoneally. The corresponding receiver (RA1010; DSI) was fixed under the animal’s cage and connected via a BMC100 consolidation matrix (DSI) to a Dataquest III computerized data analyzer (DSI). The animals were then recorded during the 6^th^ week following peptide injection and telemetric data were analyzed. This system allows measurement of continuous locomotor activity and body temperature variations, as previously reported [20].

### Spatial Short-term Memory (Delayed Alternation in the T-maze)

As previously detailed [20], [36], delayed alternation was tested in the T-maze and the results were expressed as ratio of the time spent in the initially closed novel arm over the time spent in the previous arm and as a ratio of the number of entries into the novel arm over the familiar one.

### Spatial Long-term Memory (Place Learning in the Water-maze)

As previously reported [20], [25], spatial reference memory was tested using a place learning procedure in the water-maze. Training consisted of three swims per day for 5 days. Each rat was allowed a 90 s swim to find the platform and was left for a further 30 s on the platform. The median latency was determined for each training session. A probe test was performed 4 h after the last training session. The platform was removed and each rat was allowed a free 60 s swim. The percentage of time spent in the training quadrant was determined by videotracking (Viewpoint, France).

### Endocrine Stress

Blood samples were collected after killing the rats by decapitation, as previously reported [37]. Plasma corticosterone (CORT) was assayed with a radioimmunoassay kit (Biotrak, GE-Healthcare, France) in 50 µl plasma sample diluted (1∶5) with the assay buffer. The intra- and inter-assay coefficients of variation were 5% and 7%, respectively. The assay sensitivity was 0.6 ng/ml.

### Oxidative Stress

As previously described [20], [28], quantification of lipid peroxidation in tissue extracts was based on Fe(III)xylenol orange complex formation according to the Hermes-Lima method [38].

### Specific Markers (GFAP, Iba1, VAChT, PSA-NCAM, Caspase-9, -12 and -3)

Rats were sacrificed by decapitation and structures of interest were weighed, immediately frozen in liquid nitrogen and stored at −20°C. Tissues were sonicated with a VibraCell (Sonics & Materials, USA) in 2% SDS. Homogenates were then boiled (5 min) and centrifuged for 30 min at 14000 g (for GFAP, pro- and cleaved caspase-9 and -12, and pro-caspase-3). To detect cleaved caspase-3, VAChT and Iba1, tissues were homogenized using a specific lysis buffer (Triton X100 1%; Tris-HCl pH 7.5, 20 mM; NaCl 150 mM; EDTA 10 mM; Na_3_VO_4_ 100 µM) previously described by Cotrufo et al. [39]. Supernatants were collected and the protein concentration was measured using the BCA Kit (Pierce, France) and 20 to 40 µg from each sample was taken for western blot analysis depending on the structure and antigen considered. Samples were boiled (5 min), separated by SDS-polyacrylamid gel (12%) and transferred to a nitrocellulose membrane (Whatman, France). The membrane was incubated overnight (4°C) with a mouse anti-glial fibrillary acidic protein (GFAP) (1/1000; Sigma-Aldrich, France), or a rabbit anti-Iba-1 (1/750; Wako Chemicals, Japan), or a rabbit anti-VAChT (1/500; Sigma-Aldrich) or a rabbit anti-procaspase-3 and a rabbit monoclonal anti-caspase-3 (cleaved form) (1/1000 and 1/2000, respectively; Cell Signaling, France), or a rabbit anti-caspase-9 (pro- and cleaved forms; 1/1000; Cell Signaling), or a rat anti-caspase-12 (pro- and cleaved forms; 1/5000; Sigma-Aldrich, France) or a mouse anti-β-tubulin (β-tub) (1/5000; Sigma-Aldrich). The membrane was then rinsed and incubated for 2 h with the appropriate horseradish peroxidase-conjugated secondary antibodies (Sigma-Aldrich). Peroxidase activity was revealed by using enhanced-chemiluminescence (ECL) reagents. The intensity of peroxidase activity was quantified using Image-J software (NIH, Bethesda, USA). β-tubulin was taken as loading control for all immunoblotting experiments and each value was normalized relative to respective β-tubulin level [20]. Note that in this study, the two antibodies against PSA-NCAM that were tested for western blot analysis (mouse monoclonal anti-PSA-NCAM, clone 2-2B, ref# MAB5324 from Millipore and mouse monoclonal anti-PSA-NCAM, ref# AbC0019 from AbCys SA) were unable to work in our conditions.

### APP Processing

As previously reported [20], 60 µg from each sample was taken to western blot analysis following the same procedures detailed in 2.8. The primary antibody used to detect APP (125 kDa) and C99 fragment (13 kDa) was a rabbit anti-Amyloid Precursor Protein (PA1-84165: 1/750, ABR-Thermo-Scientific, France).

### Tau Phosphorylation

To determine the levels of Tau phosphorylation at specific sites, equal amounts of protein (varying from 60 and 80 µg depending on the antibody used) from each sample were taken to western blot analysis following the same procedures detailed before. The primary antibodies to detect phospho-Tau epitopes (50 kDa) were a mouse AT8 (S^199^/S^202^/T^205^) and AT100 (T^212^/S^214^/T^217^) antibodies (MN1020: 1/3000 and MN1060: 1/3000, respectively; ThermoScientific, France), and to detect total Tau (50 kDa) was a mouse anti-Tau antibody (MA1-38710: 1/5000, ThermoScientific, France).

### BDNF Content

Rats were sacrificed by decapitation and structures of interest were weighed, immediately frozen in liquid nitrogen and stored at −20°C until assayed. Brain-derived neurotrophic factor (BDNF) content was measured with a conventional ELISA assay (BDNF Emax; Promega, France), as previously reported [40], [41]. The assay sensitivity was 15 pg/tube. The BDNF concentration was expressed as pg/g wet weight. The intra- and inter-assay coefficients of variation were 3% and 6%, respectively.

### Histology (Cresyl Violet Staining; GFAP, Iba-1, PSA-NCAM, VAChT Immunolabeling)

Animals were anaesthetized using an intramuscular injection of ketamine/xylazine solution and perfused intracardially (50 ml of NaCl 0.9% and 100 ml PO_4_ 0.2 M containing 4% paraformaldehyde). Brains were removed and postfixed in the same fixative for 48 h (4°C) and then in a solution of sucrose (30%) for 3 days. Thereafter, tissues were included in a block of OCT compound (Tissue-Tek, Sakura Finetek, USA) and quickly frozen in acetone chilled on dry ice. Frozen brains were mounted on a cryostat (Leica, France) and serially cut into 10 µm coronal sections. For histology, they were stained with 0.2% cresyl violet reagent, dehydrated and mounted. The method used for neuronal count is a classical method used with thin brain sections (10 µm) to quantify undamaged hippocampic cells [20], [29], [42], [43]. The counting was made using images captured with Leica DFC495 high-resolution camera (Leica Microsystem, Nanterre, France) attached to Leica DM2500 microscope (Leica) and the Leica LAS Core image analysis software (Leica). For this purpose, digitized images acquired using a ×40 objective were transformed into TIFF files and brought to the same level of contrast and sharpness using the software. Four sections were studied from each brain, taken from the anterior hippocampus level (−3.0 to −4.0 from the bregma) [35], with intervals of 250 µm. Sections were selected on a subjective random basis. Three fields were analyzed per hippocampus for CA1, one for CA2 and CA3 and two for DG. Counts of undamaged cells were made using ImageJ software on TIFF captured images. Neuron densities on slices (number of neurons in the optical field expressed as the number of cells per mm^2^) were calculated as the arithmetic mean number of neurons in the two hemispheres and, for each animal, as the arithmetic mean of results obtained for each of the four slices. The count of undamaged cells in the CA1, CA2, CA3 and DG fields of the hippocampus was done by two different scientists unaware of the experimental conditions and independently from each other using display projections of the images (each person performed cell count for all animals included into the experiment and no difference was observed between the two independent and unaware analyses).

Analyses of the glial (GFAP) and acetylcholine (VAChT*)* markers were conducted according to a diaminobenzidine (DAB) immunohistochemistry approach, while analyses of the microglial (Iba-1) and neurogenesis (PSA-NCAM) markers were conducted according to a fluorescent immunohistochemistry approach [20], [40]. Sections were incubated overnight at room temperature with a mouse anti-GFAP (1/1000, Sigma-Aldrich), or a rabbit anti-Iba-1 (1/500; Wako Chemicals, Japan), or a rabbit anti-VAChT (1/500; Sigma-Aldrich) or a rabbit anti-PSA-NCAM antibody (1/200; Abcys, France). Then sections were incubated for 2 h with the appropriate biotinylated (anti-GFAP and anti-VAChT) or fluorescent (Alexafluor-488; anti-Iba-1 and Alexafluor-555; anti-PSA-NCAM) secondary antibody (Sigma-Aldrich or Molecular Probes, The Netherlands). Biotinylated sections were incubated for 1 h in avidin–biotin complex (ABC kit; Vector Laboratories, USA). Signals were detected with the diaminobenzidine kit (Vector Laboratories), according to the manufacturer’s instructions, while nuclei of fluorescent sections were counterstained with 4′,6′-diamino-2-phenylindole (DAPI, Molecular Probes). The immunostaining specificity was determined with the same protocol but by incubating control sections with the secondary antiserum alone.

### Aβ_25–35_ Distribution

We used a tagged peptide with a fluorescent dye (oAβ_25–35_ HiLyte Fluor 488, ANASPEC/Eurogentec, France) (oAβ_25–35_-HLF) to analyze the distribution within brain structures of the injected oAβ_25–35_ fragment. The ability of the tagged peptide to form amyloid fibrils after 4 days of incubation at 37°C was previously assessed by electron microscopy and Congo red staining [20]. Six weeks following the icv injection of oAβ_25–35_-HLF, animals were anaesthetized using an intra-muscular injection of ketamine/xylazine solution and perfused intracardially, as previously described. Brains were removed and postfixed in the same fixative for 3 days (4°C) and serially cut with a vibrating blade microtome (Leica) into 30 µm coronal sections. Before to be mounted, sections were counterstained with DAPI to visualize nuclei.

### Statistical Analysis

Data are presented as mean±SEM. Comparisons were performed using one-way or two-way ANOVA (F values) followed by a Fisher’s multiple comparison test. P< 0.05 was considered significant.

## Results

### Regional Distribution of Aβ25–35-HLF Showed Persistent Presence of oAβ25–35 after 6 Weeks in the Brain

The regional distribution of oAβ_25–35_-HLF 6 weeks after injection is presented in Fig. 1A–I. Control rat sections were treated and examined in the same conditions as injected rat sections and displayed no specific labeling. oAβ_25–35_-HLF labeling was relatively discreet, suggesting a progressive clearance of the peptide after 6 weeks in comparison to previous study [20]. However, oAβ_25–35_-HLF was again found at the injection site level where it was trapped by local cells (Fig. 1A). The fluorescent peptide was also found in brain ventricles, particularly in the lateral ventricle (LV) (Fig. 1B–C), and at the dorsal (D3V) and ventral (3V) parts of the third ventricle (Fig. 1D–F). At this level, oAβ_25–35_-HLF was found in ependymal cells bordering ventricles and in the surrounding brain structures (Fig 1B–F). The fluorescent peptide was found in the walls of blood vessels particularly in the amygdala, frontal (Fig. 1G) and parietal cortex, but also in hypothalamus and thalamus (Fig. 1H) regions. As previously reported, in addition to ependymal cells, oAβ_25–35_-HLF was found in neurons (Fig. 1H–I) and in glial cells, particularly in the median eminence (Fig. 1E), but also in nerve fibers, principally in the hippocampus (Fig. 1I).

### 4-days Incubation of Aβ25–35 Led Almost Exclusively to Soluble Oligomers

In addition to a previous qualitative study [20], where we have evidenced the amyloid property of the aggregated Aβ_25–35_ peptide, we have determined in the present work the respective quantity of each particle species contained in the solution injected in rats. The characterization of the aggregated Aβ_25–35_ solution was realized by PCS and is presented in Figure 1J. This figure shows the size distribution of the oligomeric species in the aggregated Aβ_25–35_ solution. After 4-days incubation at 37°C, the sample is composed of particles with an average diameter of 103.4 nm (weighted average, min/max: 50.7/712.4 nm) (red curve). In order to determine the size of particles that populated the aggregated Aβ_25–35_ solution, a low-speed centrifugation was carried out at 1 000 g for 15 min. PCS analysis of the pellet resuspended in water (purple curve) indicated that the particle size extended from 295.3 to 825 nm with a peak maximum at 458.7 nm and an average diameter of 479.5 nm. For the supernatant (green curve), the weighted average diameter of particles was 60.0 nm with a peak maximum at 50.7 nm. Similar size of particles (weighted average diameter of 52.8 nm) was measured in the supernatant after centrifugation at 16 000 g (blue curve). Note that these particles had a size with at least one order of magnitude higher than the monomer form of Aβ_25–35_ peptide in HFIP (black curve). This suggests that the aggregated Aβ_25–35_ solution that has been used for icv injection (red curve) is mainly composed of a mixture of soluble oligomer species whose sizes extended from 52.8 to 295.3 nm (98.1%). Some high-density aggregates with a diameter greater of equal at 458.7 nm were also detectable but in low proportion (1.8%).

### oAβ_25–35_ Injection in Rats Failed to Affect Body Weight and Physiological Rhythms

**Figure 2:**
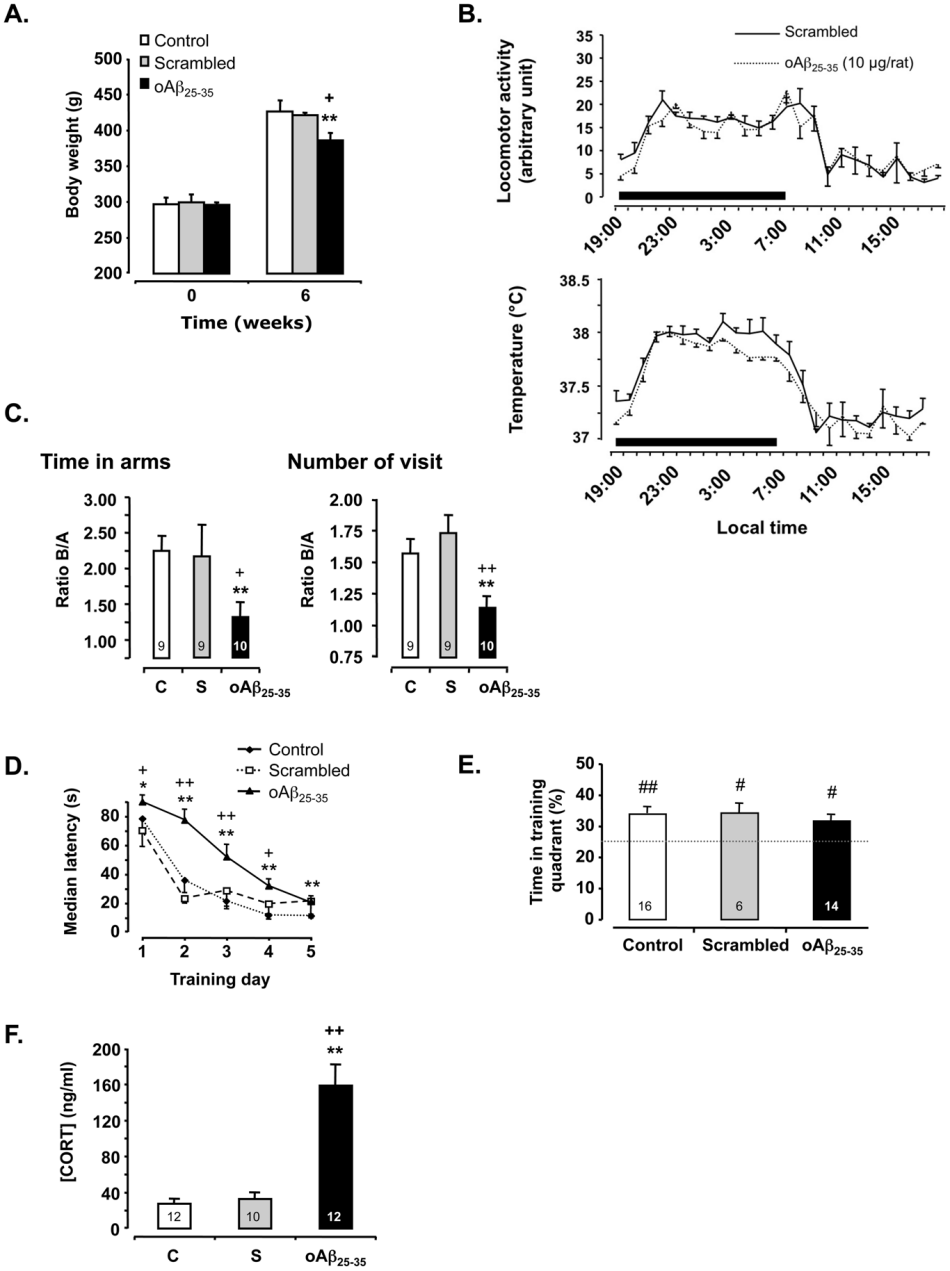
Physiological and behavioral effects of oAβ_25–35_. **A.** Body weight variations determined 6 weeks after icv injection of scrambled Aβ_25–35_ peptide (10 µg/rat; scrambled group) or oAβ_25–35_ (10 µg/rat; Aβ_25–35_ group). The results are expressed as means ± SEM (with n = 6 per group). *p<0.05 *vs*. control value and +p<0.05 vs. scrambled value). **B.** Variations in locomotor activity and body temperature determined 6 weeks after icv injection of scrambled Aβ_25–35_ peptide (10 µg/rat; scrambled group; n  = 7) or oAβ_25–35_ (10 µg/rat; oAβ_25–35_ group; n = 7). Locomotor activity and body temperature were monitored using telemetric sensors. The thick black line indicates the nocturnal phase (7:00 PM to 7:00 AM). The results are means/hour obtained the 6^th^ week following the icv injection. **C.** Effects of oAβ_25–35_ icv injection (10 µg/rat) on the ability of rats to perform a spatial short-term memory task (T-maze). Six weeks after icv injection, animals were allowed to explore the T-maze, with one short arm closed (B), for 10 min. After a 1 h time interval, the pattern of exploration of the whole maze was recorded for 2 min. The icv injection of the scrambled Aβ_25–35_ peptide (10 µg/rat) served as negative control. The results are expressed as means ± SEM. **p<0.01 *vs.* control un-injected rats, +p<0.05 and ++p<0.01 *vs.* scrambled treated rats. The number of animals in each group is indicated within the columns. **D.** Effects of oAβ_25–35_ icv injection (10 µg/rat) on rat behavior in a spatial long-term memory test (Water-maze). Six weeks after icv injection, animals were allowed to swim for 90 s to find the training platform and 60 s without the platform for retention. The icv injection of the scrambled Aβ_25–35_ peptide (10 µg/rat) served as negative control. The results are expressed as means ± SEM. *p<0.05 and **p<0.01 vs. control un-injected rats, +p<0.05 and ++p<0.01 *vs.* scrambled treated rats. **E.** The probe test was performed 4 h after the last training trial in a single 60 s-duration swimming without platform. The presence in the training quadrant was analyzed over the chance level (25%): # p<0.05 and ## p<0.01. The number of animals in each group is indicated within the columns. **F.** Variations in plasmatic corticosterone (CORT) levels determined in rats 6 weeks after icv injection of Aβ_25–35_ scrambled peptide (10 µg/rat; negative control) or oAβ_25–35_ (10 µg/rat). The values are means ± SEM. **p<0.01 *vs*. control un-injected rats (control group: C) and ++p<0.01 *vs*. scrambled treated rats. The number of animals in each group is indicated within the columns.

At the beginning of the experiment, the three groups of rats presented no body weight differences (F_2,15_ = 0.10; p>0.05) (Fig. 2A). By contrast, body weight gain was decreased 6 weeks after the icv injection of oAβ_25–35_ as compared to control and scrambled peptide-injected rats (F_2,15_ = 5.18; p<0.05), with a comparable value as previously observed [20].

Locomotor activity and body temperature rhythms were continuously recorded using telemetric transmitters and revealed only circadian differences but not among groups (Fig. 2B). In details, the two-way repeated measure ANOVA for locomotor activity and body temperature data revealed a significantly difference between night and day values but no difference induced by oAβ_25–35_ icv injection (locomotor activity: F_1,276_ = 0.13, p>0.05 for treatment; F_23,276_ = 14.5, p<0.0001 for time and F_23,276_ = 0.65, p>0.05 for interaction; body temperature: F_1,276_ = 0.73, p>0.05 for treatment; F_23,276_ = 31.2, p<0.0001 for time and F_23,276_ = 0.55, p>0.05 for interaction).

### 6 Weeks after oAβ25–35 Injection, Rats Showed Impaired Learning and Memory Capacities

The ability of rats to perform a spatial short-term memory task was examined using delayed alternation in the T-maze (Fig. 2C). The oAβ_25–35_ injection induced memory deficits, since analyses of ratios of time spent and the number of visits in the novel arm over the familiar one revealed significant effects (F_2,25_ = 8.09; p<0.01 and F_2,25_ = 3.93; p<0.05, respectively), while scrambled peptide-injected rats presented no deficits as compared to control animals (Fig. 2C).

The spatial reference memory was analyzed using a water-maze procedure. When rats started training 6 weeks after peptide injection (Fig. 2D), acquisition profiles decreased with training. Two-way repeated measure ANOVA showed an effect of training trials and a treatment effect: F_2,260_ = 28.7, p<0.0001 for the treatment, F_4,260_ = 131, p<0.0001 for trials and F_8,260_ = 3.96, p<0.001 for the interaction (Fig. 2D). These data outlined an alteration of acquisition performances. However, when animals were submitted to the probe test (Fig. 2E), oAβ_25–35_-treated rats showed a preferential presence in the training quadrant similarly as scrambled peptide-treated and control rats. The peptide injection therefore only slowed down place learning acquisition but did not impeded it.

### 6 Weeks after oAβ_25–35_ Injection, Rats Showed a Marked Endocrine Stress

The oAβ_25–35_ icv injection significantly increased plasma CORT concentrations after 6 weeks when compared with control and scrambled peptide-injected rats (F_2,31_ = 53.8, p<0.0001) (Fig. 2F).

### Analyses of Cellular Markers in oAβ25–35 Injected Rats

**Figure 3:**
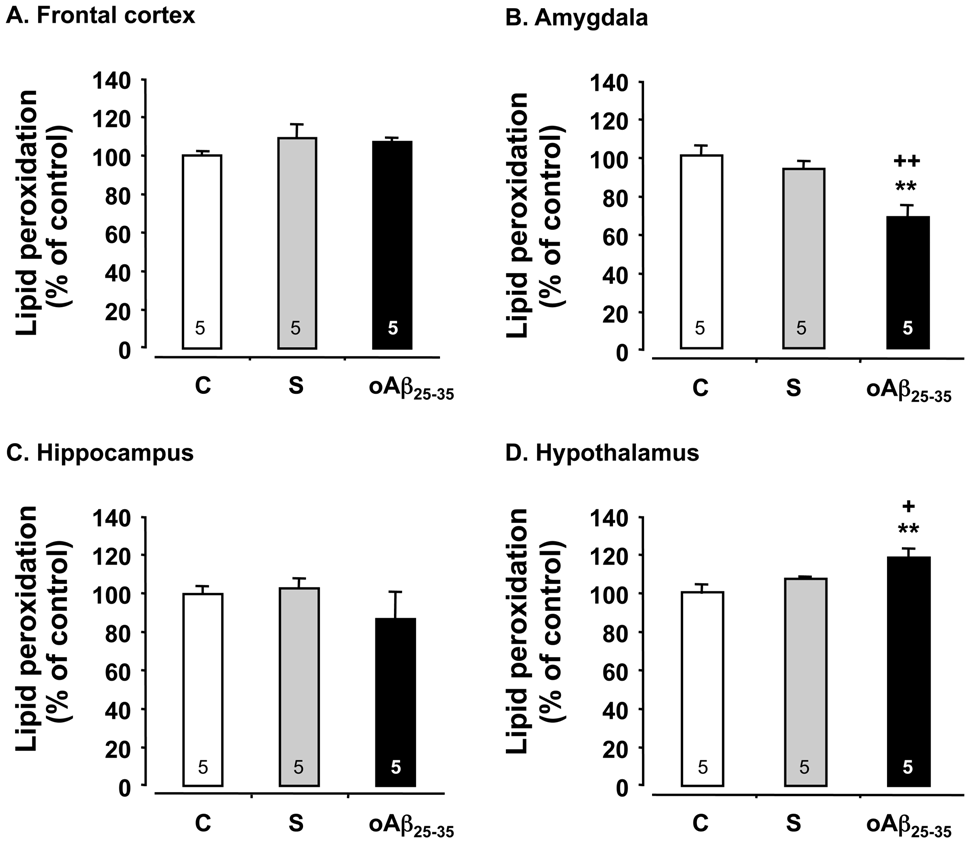
Oxidative stress. Variations in lipid peroxidation levels in the frontal cortex, amygdala, hippocampus and hypothalamus, determined in rats 6 weeks after icv injection of scrambled Aβ_25–35_ peptide (10 µg/rat; negative control) or oAβ_25–35_ (10 µg/rat). The results are expressed as means ± SEM. **p<0.01 *vs*. control un-injected rats (control group: C), +p<0.05 and ++p<0.01 *vs.* scrambled treated rats. The number of animals in each group is indicated within the columns.

When compared to control and scrambled peptide-injected rats, the oAβ_25–35_ icv injection induced after 6 weeks an increase in lipid peroxidation level in the hypothalamus (F_2,12_ = 4.95, p<0.05) (Fig. 3D), while in the amygdala, oAβ_25–35_ induced a significant decrease in lipid peroxidation levels (F_2,12_ = 17.7, p<0.001) (Fig. 3B). By contrast, no difference in peroxidized lipids was observed in the frontal cortex (F_2,12_ = 1.17, p>0.05) (Fig. 3A) and the hippocampus (F_2,12_ = 3.05, p>0.05) (Fig. 3C) in control, scrambled peptide- and oAβ_25–35_-injected rats.

**Figure 4:**
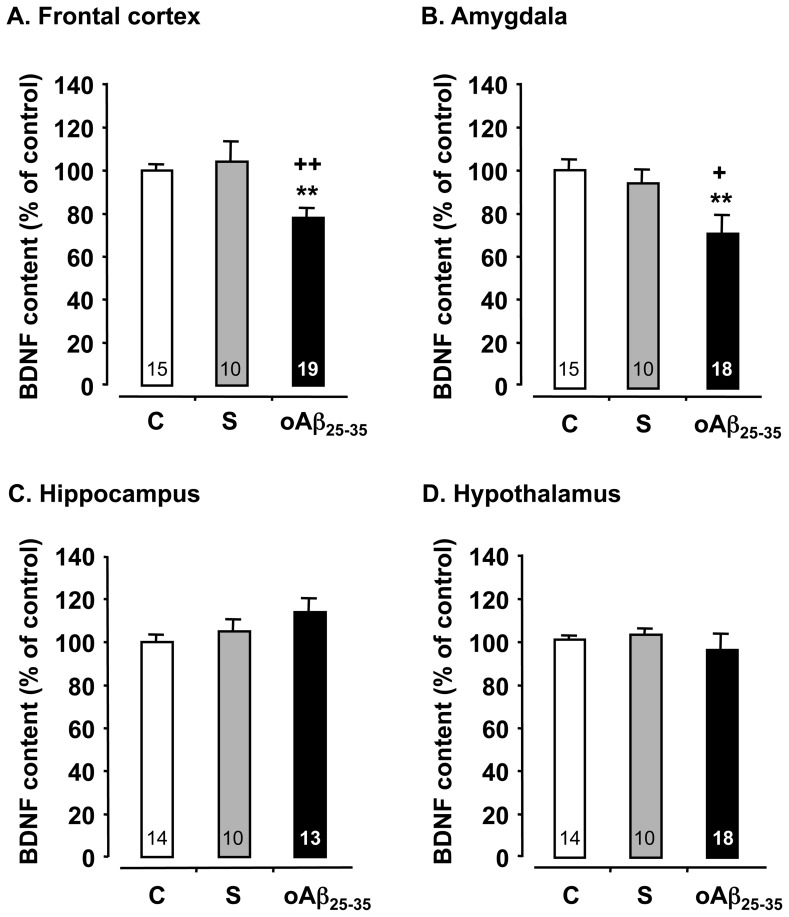
Neurotrophic factor. Variations in BDNF contents within the frontal cortex, amygdala, hippocampus and hypothalamus, determined in rats 6 weeks after icv injection of scrambled Aβ_25–35_ peptide (10 µg/rat; negative control) or oAβ_25–35_ (10 µg/rat). The results are expressed as means ± SEM. **p<0.01 *vs*. control un-injected rats (control group: C), +p<0.05 and ++p<0.01 *vs.* scrambled treated rats. The number of animals in each group is indicated within the columns.

As a major neuroprotective system, BDNF contents were analyzed in the rat brain structures. BDNF levels in the frontal cortex (Fig. 4A) and amygdala (Fig. 4B) were decreased 6 weeks after oAβ_25–35_-treated rats, when compared to control and scrambled peptide-injected rats (F_2,41_ = 3.76, p<0.05 and F_2,40_ = 5.33, p<0.01, respectively). By contrast, the oAβ_25–35_ injection induced no modification of BDNF content in the hippocampus (F_2,36_ = 1.85, p>0.05) (Fig. 4C) and hypothalamus (F_2,39_ = 1.05, p>0.05) (Fig. 4D).

**Figure 5:**
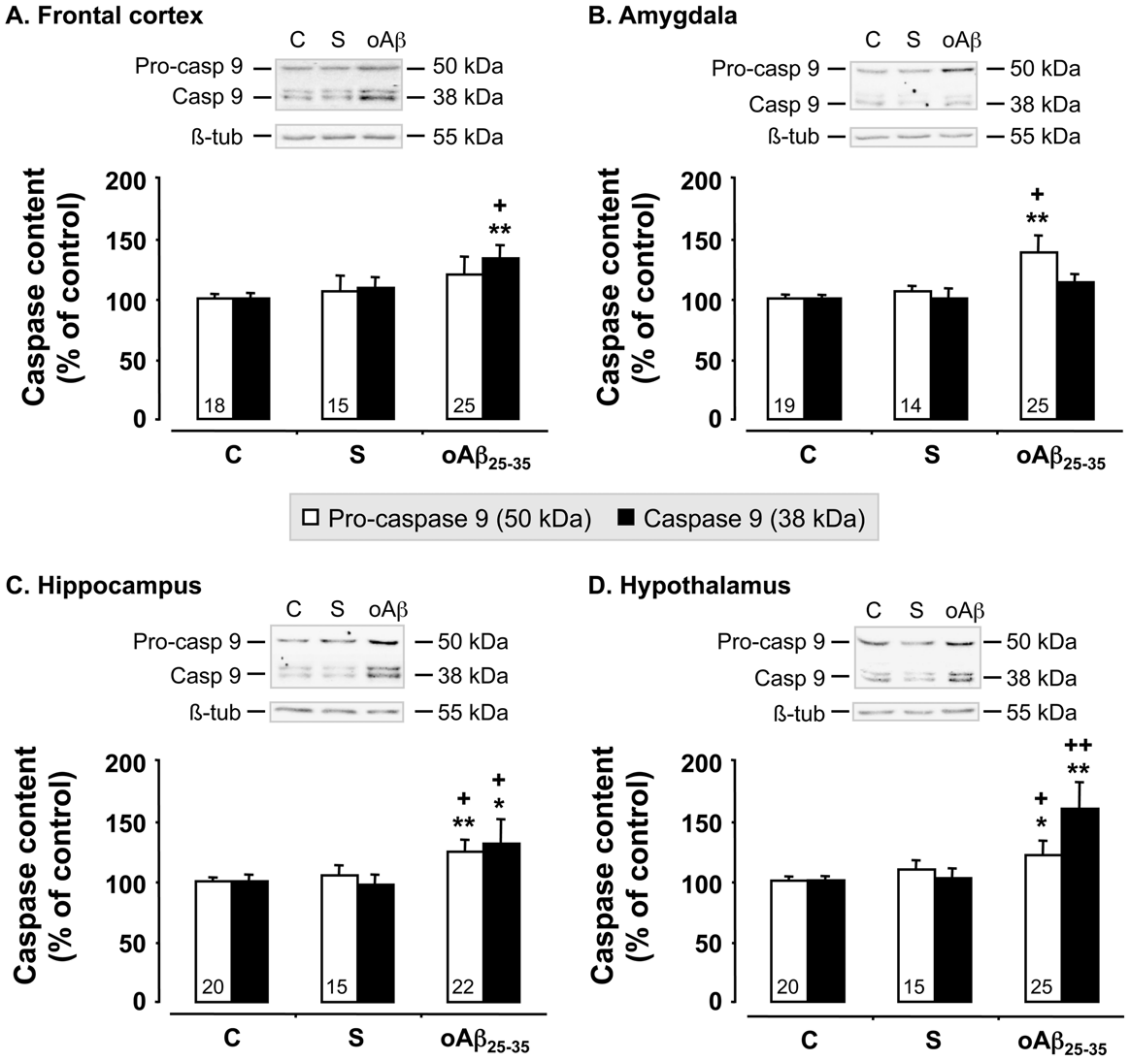
Mitochondrial stress. Pro- and activated caspase-9 levels within the frontal cortex, amygdala, hippocampus and hypothalamus, determined in rats by western blot 6 weeks after oAβ_25–35_ icv injection (10 µg/rat). Pro-caspase-9 (50 kDa) and activated caspase-9 (38 kDa) variations were normalized with β-tubulin (β-tub, 55 kDa) variations and compared with un-injected rats (control group: C). The results are expressed as means ± SEM. *p<0.05 and **p<0.01 *vs*. control group, +p<0.05 and ++p<0.01 *vs.* scrambled treated rats. Note that scrambled peptide injection (10 µg/rat) served as negative control and did not induce any modifications in pro- and activated caspase-9 levels. The number of animals in each group is indicated within the columns.

We used western blot analyses to measure the levels of pro- and activated caspase-9, an index of mitochondrial alteration, in the cerebral structures of interest, before (control rats) and 6 weeks after scrambled peptide or oAβ_25–35_ injection (Fig. 5). While scrambled peptide injection failed to affect pro- and cleaved caspase-9 levels, oAβ_25–35_ induced an increase in pro-caspase-9 levels in the amygdala (+38%; F_2,55_ = 9.25, p<0.001) (Fig. 5B), hippocampus (+25%; F_2,54_ = 5.67, p<0.01) (Fig. 5C) and hypothalamus (+21%; F_2,57_ = 3.43, p<0.05) (Fig. 5D), but not in the frontal cortex (F_2,55_ = 2.17; p>0.05) (Fig. 5A). Cleaved caspase-9 levels were increased after Aβ_25–35_ injection in the frontal cortex (+33%; F_2,55_ = 3.74, p<0.05) (Fig. 5A), hippocampus (+31%; F_2,54_ = 2.70, p<0.05) (Fig. 5C) and hypothalamus (+59%; F_2,57_ = 14.6, p<0.0001) (Fig. 5D), while no effect was observed in the amygdala (F_2,55_ = 2.08; p>0.05) (Fig. 5B).

**Figure 6:**
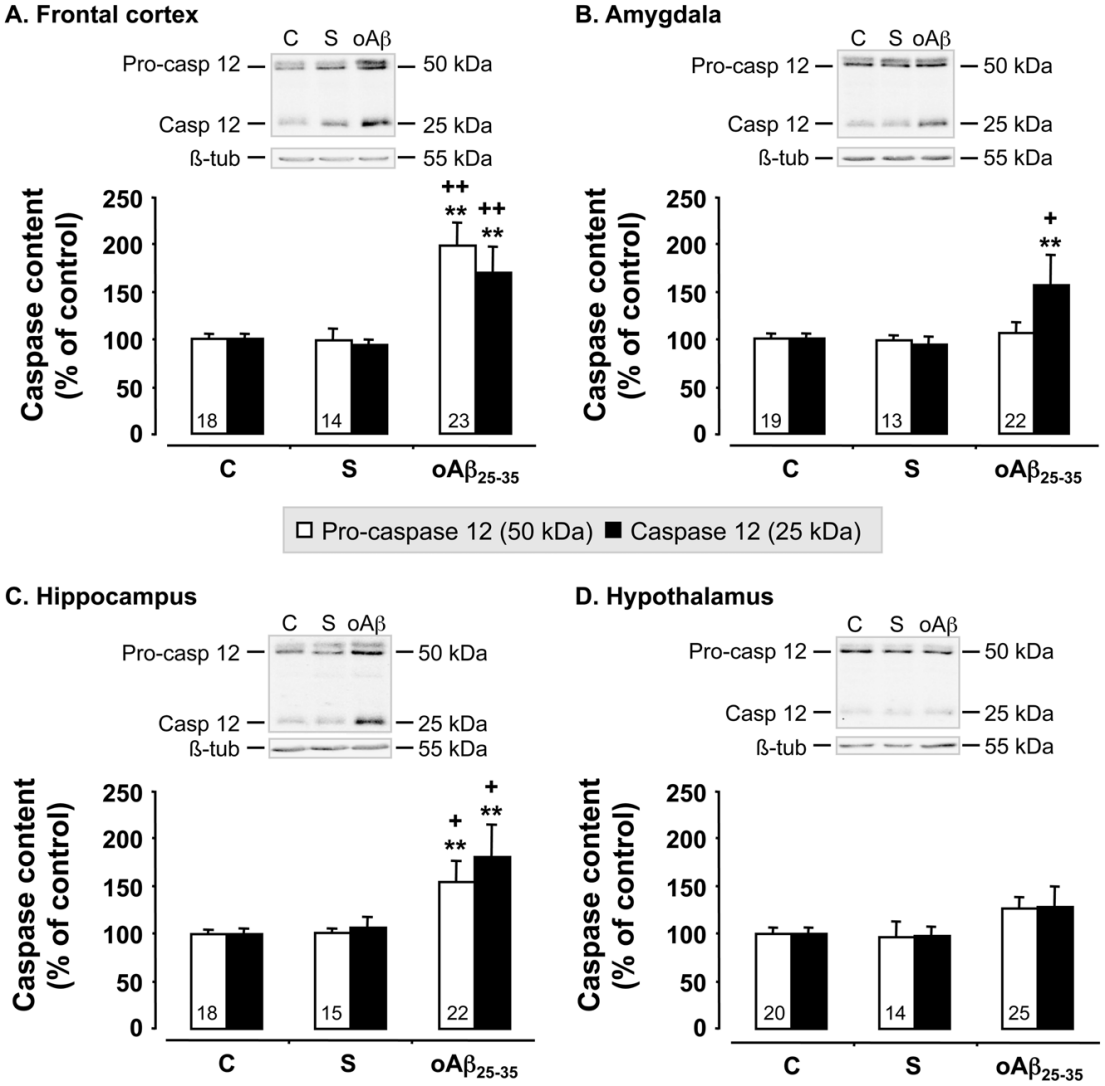
ER stress. Variations in pro- and activated caspase-12 levels in the frontal cortex, amygdala, hippocampus and hypothalamus, determined in rats by western blot 6 weeks after oAβ_25–35_ icv injection (10 µg/rat). Pro-caspase-12 (50 kDa) and activated caspase-12 (25 kDa) variations were normalized with β-tubulin (β-tub, 55 kDa) variations and compared with untreated rats (control group: C). The results are expressed as means ± SEM. *p<0.05 and **p<0.01 *vs*. control group, +p<0.05 and ++p<0.01 *vs.* scrambled treated rats. Note that scrambled peptide injection (10 µg/rat) served as negative control and did not induce any modifications in pro- and activated caspase-12 levels. The number of animals in each group is indicated within the columns.

We used western blot analyses to measure the levels of pro- and cleaved caspase-12, one of endoplasmic reticulum (ER) stress induction, before (control rats) and 6 weeks after scrambled peptide or oAβ_25–35_ injection (Fig. 6). The scrambled peptide injection failed to affect pro- and cleaved caspase-12 levels, but oAβ_25–35_ induced significant effects (Fig. 6). In detail, oAβ_25–35_ injection increased pro-caspase-12 only in the frontal cortex (+98%; F_2,52_ = 24.6, p<0.0001) (Fig. 6A) and hippocampus (+55%; F_2,52_ = 10.0, p<0.001) (Fig. 6C), while no effect was observed in the amygdala (F_2,53_ = 0.16, p>0.05) (Fig. 6B) and hypothalamus (F_2,56_ = 1.97, p>0.05) (Fig. 6D). Cleaved caspase-12 was not increased after oAβ_25–35_ injection in the hypothalamus (F_2,56_ = 1.67, p>0.05) (Fig. 6D), while a marked increase was observed in the frontal cortex (+70%; F_2,52_ = 10.9, p<0.001) (Fig. 6A), amygdala (+56%; F_2,53_ = 3.86, p<0.05) (Fig. 6B) and hippocampus (+81%; F_2,52_ = 10.8, p<0.0001) (Fig. 6C).

**Figure 7:**
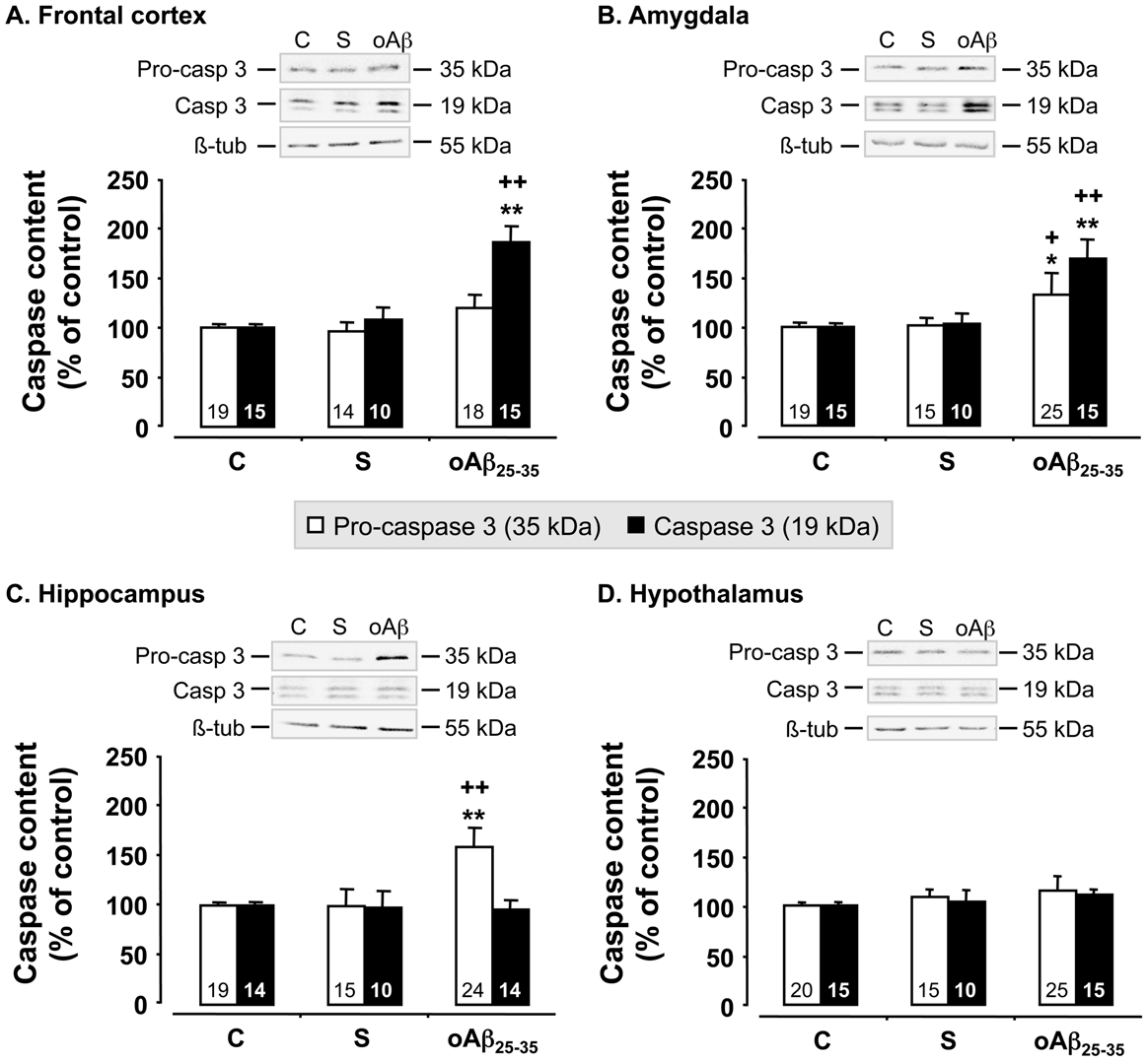
Apoptosis. Variations in pro- and activated caspase-3 levels in the frontal cortex, amygdala, hippocampus and hypothalamus, determined in rats by western blot 6 weeks after oAβ_25–35_ icv injection (10 µg/rat). Pro-caspase-3 (35 kDa) and activated caspase-3 (19 kDa) variations were normalized with β-tubulin (β-tub, 55 kDa) variations and compared with untreated rats (control group: C). The results are expressed as means ± SEM. *p<0.05 and **p<0.01 *vs*. control group, +p<0.05 and ++p<0.01 *vs.* scrambled treated rats. Note that scrambled peptide injection (10 µg/rat) served as negative control and did not induce any modifications in pro- and activated caspase-3 levels. The number of animals in each group is indicated within the columns.

We used western blot analyses to measure the levels of pro- and cleaved caspase-3, one of the apoptotic effective caspases, before (control rats) and 6 weeks after scrambled peptide or oAβ_25–35_ injection (Fig. 7). Scrambled peptide injection failed to affect pro- and cleaved caspase-3 levels, but oAβ_25–35_ induced an increase in pro-caspase-3 levels in the amygdala (+32%; F_2,56_ = 3.42, p<0.05) (Fig. 7B) and hippocampus (+59%; F_2,54_ = 17.0, p<0.0001) (Fig. 7C), but no effect in the frontal cortex (F_2,48_ = 2.84, p>0.05) (Fig. 7A) and hypothalamus (F_2,57_ = 1.70, p>0.05) (Fig. 7D). oAβ_25–35_ injection induced a marked increase in cleaved caspase-3 levels in the frontal cortex (+79%; F_2,37_ = 19.1, p<0.0001) (Fig. 7A) and amygdala (+68%; F_2,37_ = 19.1, p<0.0001) (Fig. 7B), while no effect was observed in the hippocampus (F_2,35_ = 0.03, p>0.05) (Fig. 7C) and hypothalamus (F_2,37_ = 1.54, p>0.05) (Fig. 7D).

### 6 Weeks after oAβ25–35 Injection, Astroglial and Microglial Reactions Revealed Neuroinflammation

**Figure 8:**
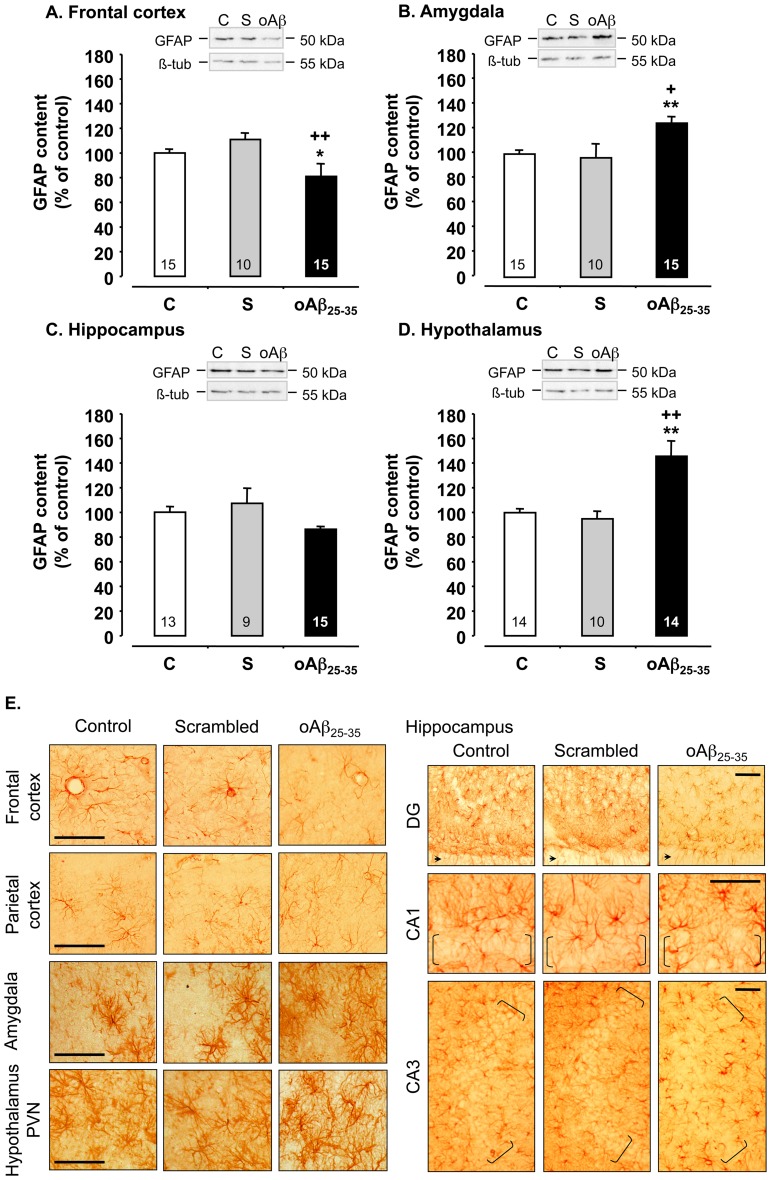
Astrocyte activation. **A.** Variations in GFAP levels in the frontal cortex, amygdala, hippocampus and hypothalamus, determined in rats by western blot 6 weeks after icv injection of scrambled Aβ_25–35_ peptide (10 µg/rat; negative control) or oAβ_25–35_ (10 µg/rat). GFAP (50 kDa) variations were normalized with β-tubulin (β-tub, 55 kDa) variations and compared with untreated rats (control group: C). The results are expressed as means ± SEM. *p<0.05 and **p<0.01 *vs*. control group, +p<0.05 and ++p<0.01 *vs*. scrambled treated rats. The number of animals in each group is indicated within the columns. **B.** Effects of oAβ_25–35_ (10 µg/rat) icv injection on astrocyte reactions using GFAP immunolabeling into the frontal and parietal cortex, amygdala, hippocampus (CA1, CA2 & CA3 regions) and hypothalamus (periventricular nucleus: PeVN & paraventricular nucleus: PVN) determined in control (C) untreated rats and 6 weeks after Aβ_25–35_ injection. The scrambled peptide injection (10 µg/rat) served as negative control and did not induce any modifications in the GFAP signal. 3v: third ventricle. brackets: hippocampus layer of granular cells layer. Scale bar  = 60 µm.

GFAP level, a marker of astroglial reactivity, was modified 6 weeks after oAβ_25–35_ injection in the frontal cortex (F_2,37_ = 3.35, p<0.05) (Fig. 8A), amygdala (F_2,37_ = 4.62, p<0.05) (Fig. 8B), and hypothalamus (F_2,35_ = 16.7, p<0.0001) (Fig. 8D), but not in the hippocampus (F_2,34_ = 1.70, p>0.05) (Fig. 8C), as compared to control and scrambled peptide icv-injected rats. In the amygdala and hypothalamus, astroglial activity increased by 25 and 46%, respectively, 6 weeks after oAβ_25–35_ injection. By contrast in the frontal cortex, GFAP level was decreased by 20%, 6 weeks after oAβ_25–35_ injection (Fig. 8A).

Increased GFAP immunoreactivity, indicative of astrogliosis was noted, essentially throughout the amygdala and hypothalamus (Fig. 8E), while no modification was observed in the parietal cortex and CA1 region of the hippocampus and a decrease was noted in the frontal cortex and other hippocampal regions (DG and CA3). Scrambled peptide icv injection did not induce any astrogliosis modification in the structures of interest (Fig. 8E).

**Figure 9:**
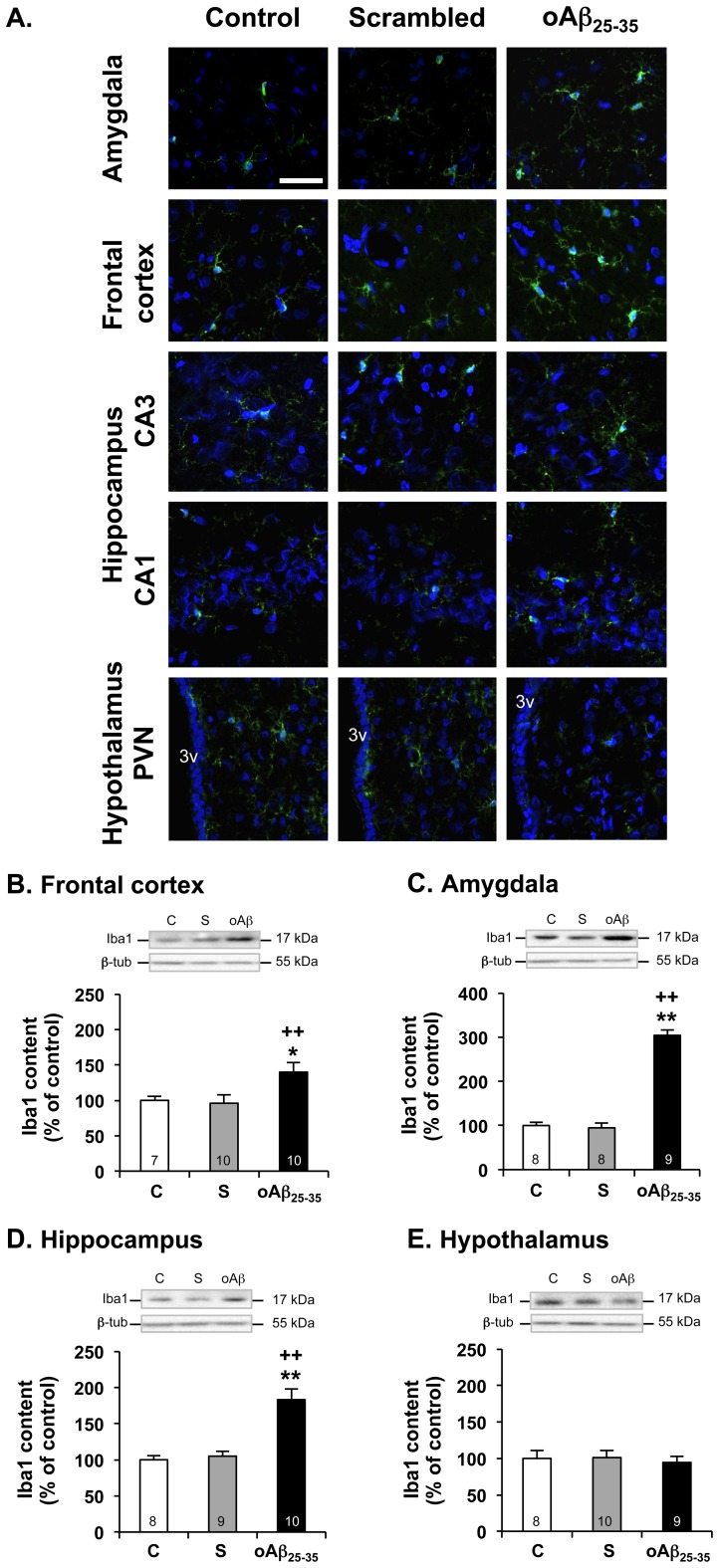
Microglial activation. **A.** Effects of oAβ_25–35_ (10 µg/rat) icv injection on microglial reaction using Iba-1 immunolabeling in the amygdala, frontal and parietal cortex, hypothalamus (paraventricular nucleus: PVN) and hippocampus (CA1 & CA3 regions) determined in control untreated rats and 6 weeks after Aβ_25–35_ scrambled peptide (10 µg/rat; negative control) or Aβ_25–35_ injection. Activated microglia was visualized with Alexafluor 488-labeled specific antibody against Iba-1 (green immunolabeling), while the nucleus was counterstained with DAPI (blue labeling). 3v: third ventricle. Scale bar  = 100 µm. **B–E.** Variations in Iba1 levels in the frontal cortex (B), amygdala (C), hippocampus (D) and hypothalamus (E), determined in rats by western blot 6 weeks after icv injection of scrambled Aβ_25–35_ peptide (10 µg/rat; negative control) or oAβ_25–35_ (10 µg/rat). Iba1 (17 kDa) variations were normalized with β-tubulin (β-tub, 55 kDa) variations and compared with untreated rats (control group: C). The results are expressed as means ± SEM. *p<0.05 and **p<0.01 *vs*. control group, +p<0.05 and ++p<0.01 *vs*. scrambled treated rats. The number of animals in each group is indicated within the columns.

Increased Iba-1 immunoreactivity, indicative of microgliosis, was essentially observed in the amygdala, frontal cortex and hippocampus 6 weeks after icv injection of oAβ_25–35_ (Fig. 9A). By contrast, no modification was observed in the hypothalamus of oAβ_25–35_ injected rats or in any of the structures considered in control and scrambled peptide icv injected rats (Fig. 9A). These immunohistochemistry observations were confirmed by western blot analysis. Indeed, microglial activity (Iba1 level) was increased 6 weeks after oAβ_25–35_ injection in the frontal cortex (F_2,23_ = 6.66, p<0.01; +40% *vs.* control) (Fig. 9B), amygdala (F_2,23_ = 146, p<0.01; +204% *vs.* control) (Fig. 9C), and hippocampus (F_2,24_ = 19.4, p<0.01; +83% *vs.* control) (Fig. 8D), but not in the hypothalamus (F_2,24_ = 0.79, p>0.05) (Fig. 8E), as compared to control and scrambled peptide icv-injected rats.

### Decrease Density of Cholinergic Neurons and Terminals was Observed 6 Weeks after oAβ_25–35_ Injection

**Figure 10:**
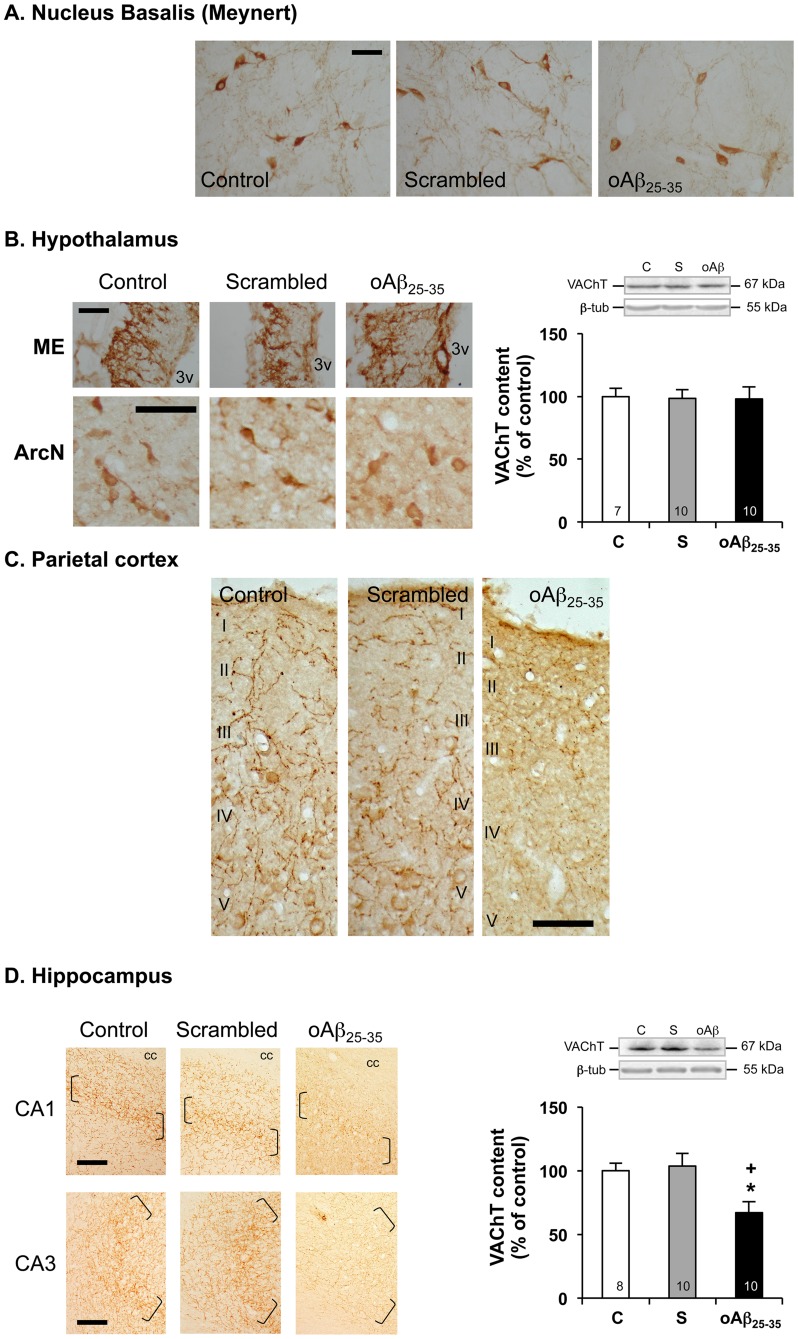
Cholinergic system. Effects of oAβ_25–35_ (10 µg/rat) icv injection on VAChT immunolabelling within the nucleus basalis of Meynert (A), mediobasal hypothalamus (B), parietal cortex (C) and hippocampus (D) determined in control untreated rats and 6 weeks after Aβ_25–35_ injection. In (B): 3v: third ventricle. In (C): levels I to V cortical layers are indicated. In (D): brackets show the hippocampus granular cell layer. cc: corpus callosum. Scale bars  = 100 µm. Variations in VAChT levels in the hypothalamus (B) and hippocampus (D), determined in rats by western blot 6 weeks after icv injection of scrambled Aβ_25–35_ peptide (10 µg/rat; negative control) or oAβ_25–35_ (10 µg/rat). VAChT (70 kDa) variations were normalized with β-tubulin (β-tub, 55 kDa) variations and compared with untreated rats (control group: C). The results are expressed as means ± SEM. *p<0.05 and **p<0.01 *vs*. control group, +p<0.05 and ++p<0.01 *vs*. scrambled treated rats. The number of animals in each group is indicated within the columns.

In control rats, large numbers of VAChT-positive cell bodies were seen in the nucleus basalis (Meynert) (Fig. 10A). In the hypothalamus, a very dense plexus of VAChT immunoreactive fibers were present in the external layer of the median eminence and weakly VAChT-positive cell bodies were noted in the arcuate nucleus (Fig. 10B). A dense network of VAChT-positive nerve fibers was seen in the parietal cortex, with the highest density in layers I, IV and V (Fig. 10C). In the hippocampal formation (Fig. 10D), the highest density of VAChT-positive fibers was seen in the pyramidal cell layer of CA1-CA3 regions. No modification was observed after scrambled peptide icv injection. By contrast, oAβ_25–35_ icv injection appeared to induce a pronounced decrease in VAChT immunolabeling in the nucleus basalis (Fig. 10A), parietal cortex (Fig. 10C) and hippocampus (Fig. 10D), while no effect seemed to be induced by oAβ_25–35_ injection in the hypothalamus (Fig. 10B). These immunohistochemistry observations were confirmed by western blot analysis particularly in well-defined structures, i.e. hypothalamus (Fig. 10B; F_2,24_ = 0.02, p>0.05) and hippocampus (Fig. 10D; F_2,25_ = 5.59; p<0.01; −23% *vs.* control).

### Hippocampus Integrity was also Impaired 6 Weeks after oAβ_25–35_ Injection

**Figure 11:**
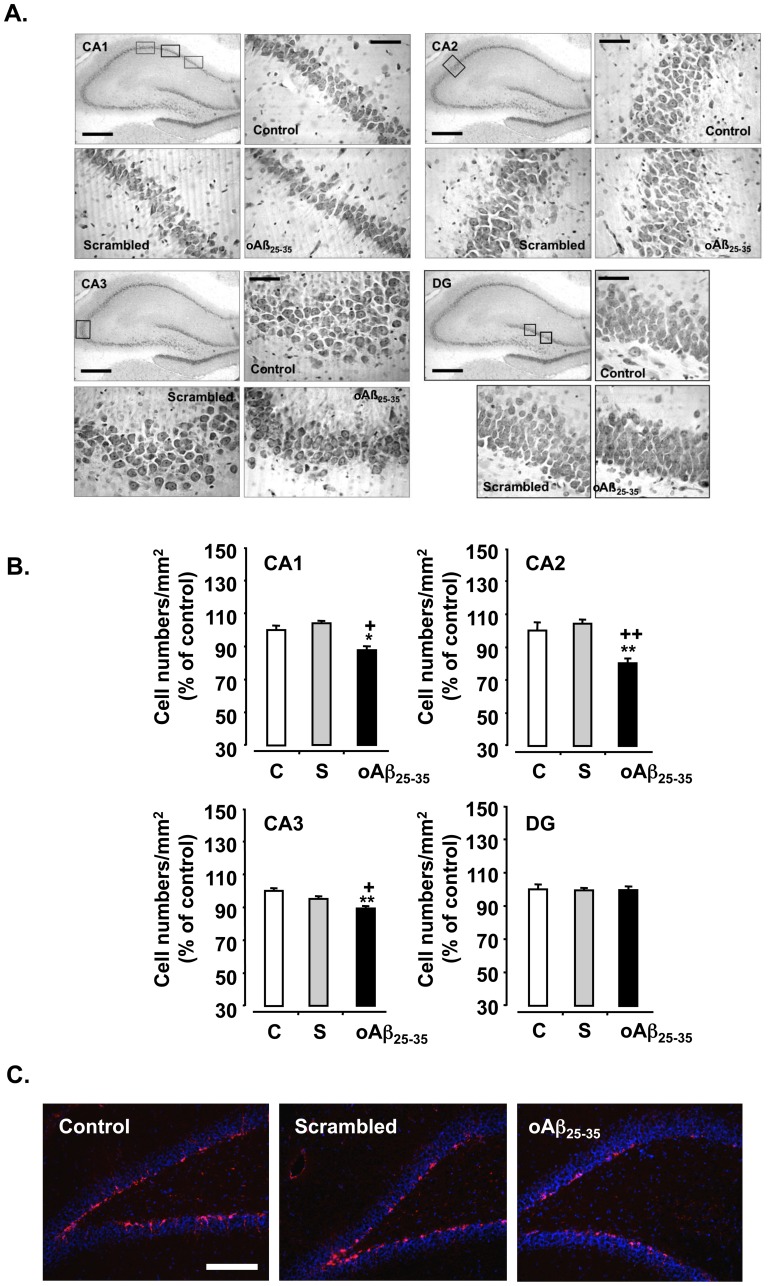
Hippocampus integrity. Variations in hippocampus pyramidal cell numbers determined in rats 6 weeks after icv injection of scrambled Aβ_25–35_ peptide (10 µg/rat; negative control) or oAβ_25–35_ (10 µg/rat). **A.** Representative microphotographs of coronal sections of Cresyl violet stained hippocampus CA1, CA2, CA3 and DG subfields, obtained in control untreated rats and after scrambled Aβ_25–35_ peptide or Aβ_25–35_ icv injection. Scale bar  = 300 and 100 µm. **B.** Average numbers of hippocampus pyramidal cells determined in untreated control rats (C) and 6 weeks after icv injection of scrambled Aβ_25–35_ peptide (10 µg/rat; negative control) or oAβ_25–35_ (10 µg/rat). The results are expressed as means ± SEM (with n  = 4 per group). *p<0.05 and **p<0.01 vs. control rats, +p<0.05 and ++p<0.01 vs. respective scrambled peptide-treated rats. **C.** Effects of oAβ_25–35_ (10 µg/rat) icv injection on hippocampus dendate gyrus (DG) neurogenesis using PSA-NCAM immunolabeling determined in untreated control rats and 6 weeks after Aβ_25–35_ scrambled amyloid peptide (10 µg/rat; negative control) or oAβ_25–35_ injection. Neurogenesis was visualized within coronal sections of the DG with Alexafluor555-labeled specific antibody against PSA-NCAM (red immunolabeling), while the nucleus was counterstained with DAPI (blue labeling). Scale bars = 200 µm.

Loss of pyramidal cells in the hippocampus was determined using Cresyl violet staining before (control) and 6 weeks after scrambled peptide or oAβ_25–35_ injection (Fig. 11A). No modification was observed after scrambled peptide injection. By contrast, oAβ_25–35_ injection induced a decrease in stained cells in the CA1 (−12%; F_2,9_ = 6.76, p<0.05), CA2 (−16%; F_2,9_ = 13.2, p<0.01) and CA3 (−10%; F_2,9_ = 14.1, p<0.01) hippocampus subfield, while no modification was observed in the DG (F_2,9_ = 0.58, p>0.05), when compared to control and scrambled peptide-injected rats (Fig. 11B).

PSA-NCAM-positive cells, indicative of neurogenesis, were revealed within the DG. An apparent decline in PSA-NCAM immunolabeling was observed 6 weeks after oAβ_25–35_ injection, while no modification was induced by scrambled peptide icv injection (Fig. 11C).

### oAβ_25–35_ Modified APP Processing 6 Weeks after Injection

**Figure 12:**
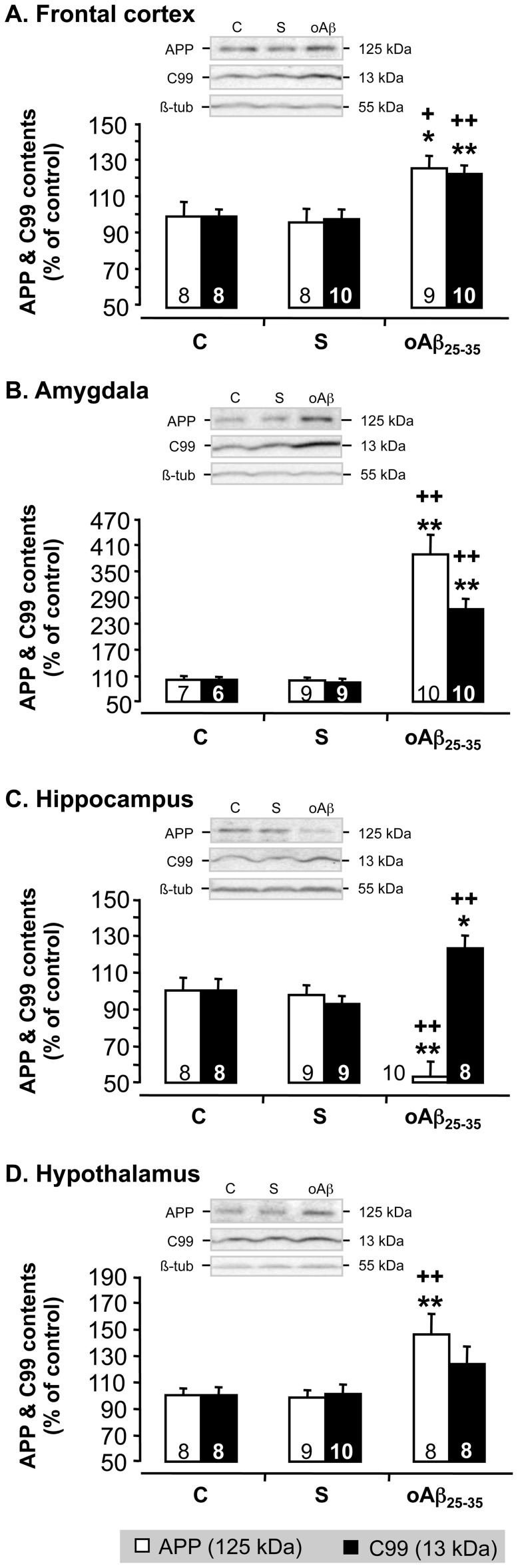
APP processing. Effects of oAβ_25–35_ (10 µg/rat) icv injection on APP processing in the frontal cortex, amygdala, hippocampus and hypothalamus, determined by western blot in untreated control rats and 6 weeks after Aβ_25–35_ scrambled amyloid peptide (10 µg/rat; negative control) or oAβ_25–35_ injection. APP (125 KDa) and C99 (13 KDa) variations were normalized with β-tubulin (β-tub, 55 KDa) variations and compared with non-injected rats (control group: C). The results are expressed as means ± SEM. *p<0.05 and **p<0.01 vs. control non-injected rats (control group: C) and +p<0.05 and ++p<0.01 vs. respective scrambled peptide-treated rats. The number of animals in groups is indicated within the columns.

To determine whether the oAβ_25–35_ injection impacted APP processing, we measured APP and C99 levels by western blot (Fig. 12). The scrambled peptide injection did not affect APP and C99 levels. By contrast, oAβ_25–35_ injection provoked a sustained increase in APP levels after 6 weeks in the frontal cortex (Fig. 12A, F_2,22_ = 5.95; p<0.01), amygdala (Fig. 12B, F_2,23_ = 30.2; p<0.01) and hypothalamus (Fig. 12D, F_2,22_ = 7.56; p<0.01), while in the hippocampus Aβ_25–35_ injection induced a significant decrease in APP levels (Fig. 12C, F_2,24_ = 13.6; p<0.01). In all structures considered, oAβ_25–35_ injection provoked a sustained increase in C99 levels, which was significant in the cortex frontal (F_2,25_ = 8.52; p<0.01), amygdala (F_2,22_ = 31.3; p<0.01) and hippocampus (F_2,22_ = 7.53; p<0.01), but not in the hypothalamus (F_2,23_ = 2.01; p>0.05). In comparison of all other structures analyzed, in the amygdala the increase of APP and C99 levels observed 6 weeks after the Aβ_25–35_ injection was extremely marked (× 3.8 and × 2.6, respectively) (Fig. 12B).

### Tau Phosphorylation was also Altered 6 Weeks after oAβ_25–35_ Injection

**Figure 13:**
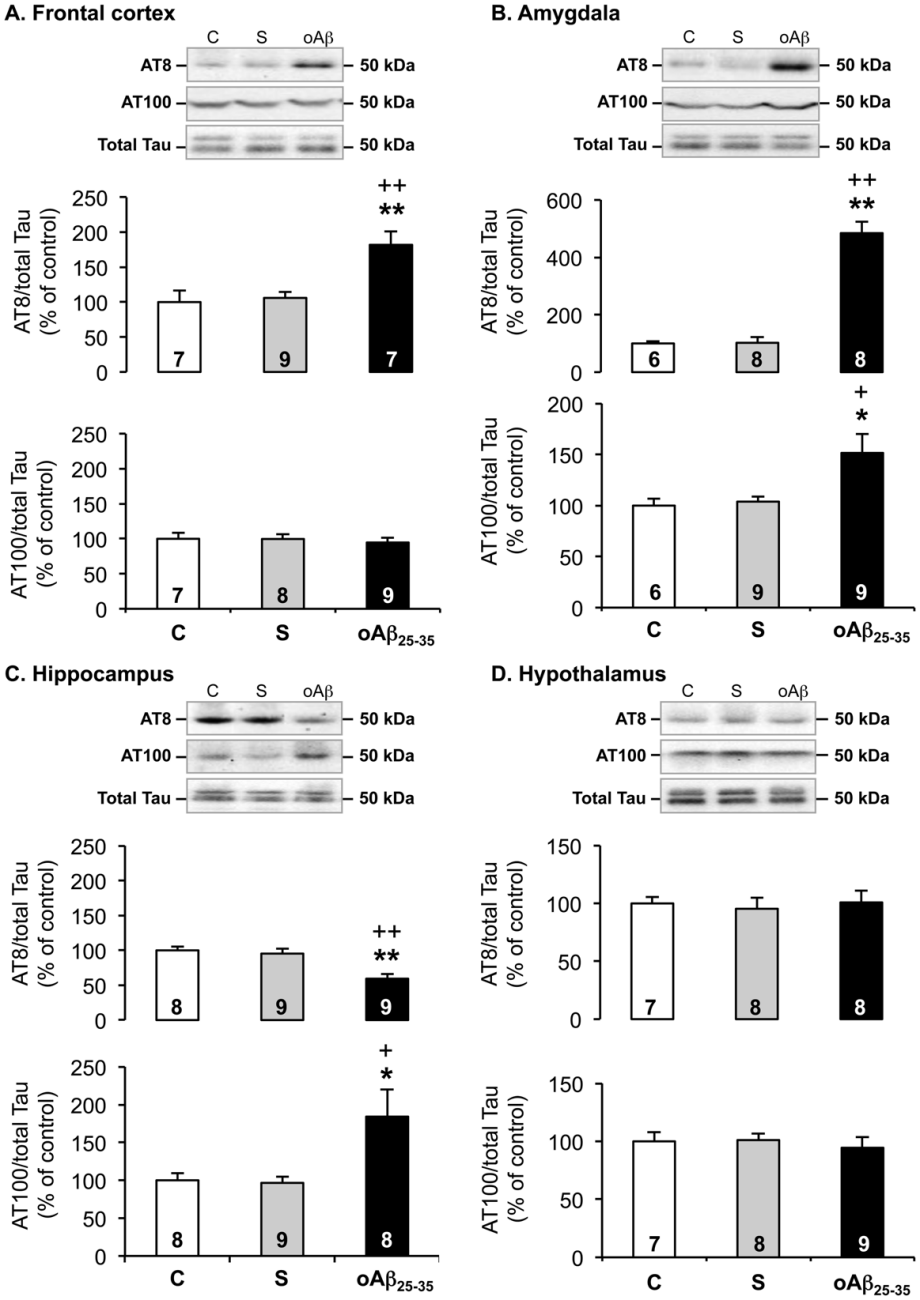
Tau phosphorylation. Effects of oAβ_25–35_ (10 µg/rat) icv injection on Tau phosphorylation in the frontal cortex, amygdala, hippocampus and hypothalamus, determined by western blot in untreated control rats and 6 weeks after Aβ_25–35_ scrambled amyloid peptide (10 µg/rat; negative control) or oAβ_25–35_ icv injection. The Tau hyperphosphorylation (AT8; 50 KDa) and the abnormal Tau phosphorylation (AT100; 50 KDa) variations were expressed in function of total Tau expression (50 KDa) and compared with non-injected rats (control group: C). The results are expressed as means ± SEM. *p<0.05 and **p<0.01 vs. control non-injected rats (control group: C) and +p<0.05 and ++p<0.01 vs. respective scrambled peptide-treated rats. The number of animals in groups is indicated within the columns.

To determine whether the oAβ_25–35_ injection altered after 6 weeks the phosphorylation level of Tau, we measured AT8, AT100 and total Tau levels by western blot. The results were then expressed as phospho-Tau/total Tau ratios (Fig. 13). The scrambled peptide injection did not affect the phosphorylation of AT8 and AT100 epitopes (Fig. 13). By contrast, oAβ_25–35_ injection provoked a sustained increase of Tau phosphorylation (AT8) in the frontal cortex (Fig. 13A, F_2,20_ = 9.24; p<0.01) and amygdala (Fig. 13B, F_2,19_ = 61.0; p<0.01). In the hypothalamus (Fig. 13D), no effect was observed (F_2,20_ = 0.11; p>0.05). In the hippocampus, a significant decrease was evidenced (Fig. 13C, F_2,23_ = 11.0; p<0.01). The AT100 phosphorylation was significantly increased only in the amygdala (Fig. 13B, F_2,21_ = 5.84; p<0.01) and hippocampus (Fig. 13D, F_2,22_ = 5.33; p<0.01). No modification was observed in the frontal cortex (Fig. 13A, F_2,21_ = 0.19; p>0.05) and hypothalamus (Fig. 13C, F_2,21_ = 0.23; p>0.05).

## Discussion

### Long-term AD-like Toxicity after oAβ25–35 Peptide Injection in Rats

The main finding of this study is that a single icv injection of oAβ_25–35_ provoked important physiological, behavioral, biochemical and morphological alterations 6 weeks after injection. The results revealed a clear similarity with numerous relevant signs of the pathology and were in line with the amyloid cascade hypothesis, while also suggesting the possible involvement of soluble oligomeric Aβ fragments in the etiology of AD [3], [44].

The long-term effects of oAβ_25–35_ seemed to be generalized since the peptide injection provoked measurable short- and long-term memory deficits, hypersecretion of glucocorticoids, BDNF deficit in the frontal cortex and amygdala, modification of APP processing and Tau phosphorylation, alteration of ER and mitochondrial homeostasis, apoptosis in the frontal cortex and amygdala, neuroinflammation processes, cholinergic deficits, hippocampal cell loss and an apparent decrease in hippocampal neurogenesis. Inflammation was maintained through astroglial and/or microglial activation in all structures considered. This observation is in accordance with AD hallmarks [45]–[49], insofar as neuroinflammatory processes were observed in AD patients at all pathological stages, processes which could participate in the amplification of the Aβ peptides-induced toxicity [50]–[53].

The impact of oAβ_25–35_ on cholinergic neurons observed at earlier stages [20] was maintained after 6 weeks. VAChT immunoreactivity was decreased in the hippocampus, parietal cortex and basal nuclei of Meynert, but not in the hypothalamus. The cholinergic deficits induced by oAβ_25–35_ injection therefore seemed consistent with the well-characterized pathological hallmarks described in AD [54], [55]. An effect that could be explained in part by the high levels of circulating glucocorticoids evidenced here that was shown to increase Aβ_1–42_ and NMDA-induced neurodegeneration in cholinergic neurons from the nucleus basalis in rat [56].

In the hippocampus, oAβ_25–35_ icv injection induced cellular loss after 6 weeks, but only in pyramidal cells of all CA regions, while no effect was observed in granular cells of DG. In a prior study, we showed an early cellular loss in the DG [20]. This particular difference between each hippocampus region evidenced over the time deserves a very precise quantification using a stereological approach, but it could be explained in part by neurogenesis modifications between the 3^rd^ and 6^th^ week following oAβ_25–35_ injection. PSA-NCAM immunolabeling seems to be more markedly decreased during the first weeks after injection than observed here after 6 weeks. This interesting observation that DG neurogenesis could finally be able to mitigate hippocampus cell loss must be accurately characterized including proliferation, migration and maturation of newborn cells with adequate markers [57], as previously reported between 5 to 30 days by Li and Zuo [58]. In other hippocampal regions, several hypotheses must be considered. Since glucocorticoids act synergistically with excitatory amino acids, particularly with glutamate [59], [60], chronic overstimulation could be extremely toxic [61]. Indeed, a hypersecretion of glucocorticoids is observed 6 weeks after oAβ_25–35_ injection at levels that could effectively results in a deleterious effect of glucocorticoids. No other data is available at present on the effect of Aβ peptide injection on glucocorticoid regulation. However, several studies demonstrated that glucocorticoids modulated APP processing [62], increased, as previously described, Aβ_1–42_- and NMDA-induced neurodegeneration in cholinergic neurons from the nucleus basalis in rat [56] and Aβ_25–35_ toxicity in hippocampus neurons [63]. These observations are also coherent with AD symptoms, since a hypersecretion of glucocorticoids was frequently observed in AD patients [64], [65].

The hippocampus cell loss is likely the result of apoptotic processes. An increase in pro- and cleaved forms of caspase-9 and -12 was observed in the hippocampus, which reflects mitochondrial and ER stress. However, these increases in initiator caspases were not associated with an activation of the effector caspase, caspase-3. Since initiator caspase-9, which can be induced by caspase-12 [66]–[68], also activates caspase-6 [69], it is possible that the cell loss in the hippocampus could be in part due to an activation of caspase-6 in this model. As an alternative, necrosis cell death could also be envisaged since, *in vivo*, the complete elimination of apoptotic cells prevents an inflammatory response, whereas necrosis often results in inflammatory reactions [70]. Moreover, in cell culture models, oAβ_25–35_ was reported to induce apoptosis at lower concentrations (5, 10 µmol/l) and necrosis at higher concentrations (20, 40 µmol/l) [71]. In the frontal cortex and amygdala, oAβ_25–35_ injection induced BDNF deficits after 6 weeks. These deficits were in line with previous results, since BDNF deficits were already observed from 2 weeks in the frontal cortex and 3 weeks in the amygdala [20]. In addition, a significant increase in caspases 9, 12 and 3 was observed in these two structures 6 weeks after oAβ_25–35_ injection. As previously reported in several studies [72]–[74], BDNF deficits could be associated with a decrease in survival pathways and therefore participate in the initiation of apoptotic processes. The expression of BDNF receptors, TrkB isoforms and p75, and other neurotrophins, particularly nerve growth factor (NGF), must therefore be analyzed to clarify the involvement of trophic factors in the toxic effects induced by oAβ_25–35_ injection. Consequently, the oAβ-induced toxicity, notably apoptotic processes, hippocampic cell loss, glucocorticoids increase, BDNF impairment and cholinergic deficits came with and could be considered as responsible for the delayed learning and memory impairments observed in oAβ_25–35_ treated rats.

It must be noted that the oAβ_25–35_ effects measured here on various markers led to variations that could appear as not correlated within a particular brain region, particularly at the 6 weeks time-point used in the present study. These often subtle changes are likely due to the particular time-course of the toxicity and intrinsic vulnerability of each brain structure, as previously reported at shorter times [20] and as observed in the pathology [75]. For instance, the hippocampus that is precociously damaged after oAβ_25–35_ [20], shows in the present work, after 6 weeks, some signs of recovery in terms of lipid peroxidation, BDNF levels, or inflammation. In the amygdala, the lipid peroxidation decrease observed 6 weeks after oAβ_25–35_ was in the continuity and very coherent with the measures made at earlier times in this brain region [20]. Indeed, this apparent sign of recovery could be simply explained by the fact that after the massive and rapid oxidative stress observed during the 1^st^ and 2^nd^ weeks after icv injection, endogenous neuroprotective mechanisms operate, limiting the extension of lipid peroxidation by increasing activity of the enzymes involved in clearance of peroxidized lipids. Such enzymes include glutathione peroxidase activity that has been recently reported to be modified after Aβ_25–35_ [76], [77]. Moreover, while in our previous study the hypothalamus appeared to be relatively insensitive to oAβ_25–35_ [20], it seemed, here, that the effects induced by the amyloid peptide progressively reached this structure. Indeed, an increase in oxidative stress in the hypothalamus was shown, correlated with an increase in caspase-9 levels. Clearly, oAβ_25–35_ induced mitochondrial dysfunction in the hypothalamus associated with neuroinflammation processes and an increase of APP level after 6 weeks. However, no effect of oAβ_25–35_ was observed on hypothalamic cholinergic neurons, Tau phosphorylation, C99 levels and caspase 12 and 3 levels. Likewise, in *post-mortem* studies, amyloid deposits were detected in the hypothalamus in late phases of AD [78]. All Aβ plaques in the hypothalamus were of the Congo red-negative amorphic type [79], [80] and comparable to the morphology of amyloid deposits observed in hippocampal and cortical structures more precociously in AD [81]. These observations show that Aβ accumulation in the hypothalamus is a delayed event compared to other structures in AD. In our model, hypothalamus seemed to show, within the time scale used in this study, a similar pattern, since it was necessary to wait 6 weeks to observe the consequences of oAβ_25–35_ toxicity in this structure. These delayed effects of oAβ_25–35_ could be partly due to a return of BDNF at basal levels after a sustained increase observed before [20]. As previously discussed, a decrease in BDNF-dependent survival pathways could facilitate oAβ_25–35_ toxicity in this structure.

### Involvement of Soluble Aβ Short Fragments in the AD Physiopathology

For the first time in this study, we have characterized the particles composition of the aggregated Aβ_25–35_ solution injected icv in rats. Indeed, while in previous study [20] we detailed qualitatively each component of the injected solution, here we showed that the majority of particles (more than 98%) were small soluble particles, suggesting that the toxicity observed after the icv injection of this peptide (oAβ_25–35_) could be due to a mixture of soluble oligomers. However, in a previous study [25], we have showed that the injection of a non-aggregated solution of Aβ_25–35_ induced less toxicity than the aggregated solution, suggesting that bigger particles would be necessary to the toxicity. This hypothesis is reinforced by a previous result using an electronic microscopy approach and showing that bigger particles (amyloid fibrils) seemed to stabilize the smaller soluble particle forms [20].

In addition, the long-lasting presence of oAβ_25–35_-HLF tagged peptide in the brain not only showed that such short fragments have *in vivo*, an important lifespan within brain tissues, but also strongly suggested that they could participate in the maintenance of the toxicity as observed here.

Six weeks after oAβ_25–35_ injection, APP processing, and particularly the amyloidogenic pathway (C99 levels), was increased in the frontal cortex, amygdala and hippocampus. This long-term effect of oAβ_25–35_ could contribute to the global toxicity always observed after 6 weeks. Interestingly, the increase in C99 level is accompanied with a decrease in APP level in the hippocampus. In the other structures examined, both APP and C99 levels were concomitantly increased. This difference could be due to a specific activation of BACE in the hippocampus, as previously suggested [82], while, in the other structures, oAβ_25–35_ could only induce an increase in APP expression and processing. Furthermore, as previously discussed [56], [62], [63], the relation between the high corticosterone levels induced by oAβ_25–35_ and the activation of amyloidogenic pathway must therefore be further analyzed to clarify the contribution of glucocorticoids deregulation in the amyloid toxicity and more largely in AD etiology.

oAβ_25–35_ injection also modified Tau phosphorylation. Previous studies showed an increase of Tau phosphorylation up to 3 months after intra-amygdala injection of Aβ_25–35_ or 4 weeks after icv injection [32], [83]. In these studies, the authors did not perform the distinction between the different phosphorylation epitopes of Tau. Here, we used two antibodies directed against AT8 and AT100 epitopes, both considered as markers of AD-related Tau phosphorylation [84]. We evidenced clearly a difference of sensitivity to oAβ_25–35_ among the brain regions considered. An increase of AT8 phosphorylation was noted only at the frontal cortex and amygdala levels, while it was decreased in hippocampus and unchanged in the hypothalamus. Concomitantly, in the same rats, the AT100 phosphorylation was increased in the amygdala and hippocampus and unchanged in the frontal cortex and hypothalamus. In fact, one explanation of these differences of sensitivity to amyloid peptide comes from recent study, where the authors evidenced an intrinsic specific regulation of Tau phosphorylation. Indeed, it seems that Tau phosphorylation occurred in a sequential order of events and that feedback mechanisms exist within neurons where the phosphorylation of certain sites would induce the dephosphorylation of other sites, in order to constantly maintain a phosphorylation level [85]. Thus in our study, it could be suggested that each brain regions could be at a different stage of Tau phosphorylation, and for instance at the hippocampal level that the phosphorylation decrease observed of the AT8 epitope could be under a negative feedback loop exerted by the phosphorylation of the AT100 epitope shown in this structure 6 weeks after oAβ_25–35_ injection.

There is no doubt that progressive Aβ accumulation contributes to the AD pathology and that extracellular amyloid deposits are a hallmark of AD. However, the view that the mature amyloid fibril was the only toxic species of Aβ is now challenged. Indeed, experimental studies have shown for a range of peptides and proteins that amyloid fibril formation is preceded by the appearance of organized molecular assemblies, usually termed protofibrils [86]. In addition, detailed biophysical studies are currently identifying the formation of smaller oligomeric species at earlier stages of the aggregation process, *in vitro*, in animal models and in patient brains. In fact, it appears highly conceivable that amyloid deposits may only be one aspect of a larger pathological cascade and indirect consequences of protective responses geared towards sequestering toxic soluble Aβ molecules within plaques, from which oligomeric toxic fragments could be released by proteolysis [4], [9], [10], [87]. The peculiar potent aggregative ability and neurotoxicity of oAβ_25–35_, its capability to induce *de novo* the Aβ_1–40/42_ protein synthesis and the abnormal phosphorylation of Tau, now even discovered physiologically to be present in elderly people [14]–[16], reinforce its potential involvement in the pathogenesis of AD.

In summary, we showed that a single icv oAβ_25–35_ injection resulted in long-term biochemical, morphological and behavioral alterations, suggesting a progressive evolution of oAβ_25–35_ deleterious effects and reinforced the face validity to this non-transgenic model of AD. This study also provided further insight on the pathological role of oligomeric Aβ fragments with a higher β-sheet potential [12]–[16], [18], [20], which could have been largely underestimated, as recently reviewed [87]. Remarkably, this fragment resulting from Aβ_1–40_ and Aβ_1–42_ proteolysis appears extremely toxic, with an important lifespan in brain tissues and could significantly contribute to the overall toxicity and therefore to the maintenance of the progressive neurodegeneration processes observed in AD, through particularly an inhibition of BDNF, an increase of apoptotic processes, a glucocorticoid hypersecretion, and the induction of the amyloid pathway and abnormal phosphorylation of Tau.
